# Frequency of resistance training does not affect inhibitory control or improve strength in well-trained young adults

**DOI:** 10.1371/journal.pone.0206784

**Published:** 2018-11-02

**Authors:** Leonardo S. Fortes, Manoel C. Costa, Maria E. C. Ferreira, José R. A. Nascimento-Júnior, Lenamar Fiorese, Dalton R. A. A. Lima-Júnior, Edilson S. Cyrino

**Affiliations:** 1 Graduate Program in Physical Education, Federal University of Pernambuco, Recife, Brazil; 2 Superior School of Physical Education, Pernambuco University, Recife, Brazil; 3 Graduate Program in Physical Education, Federal University of Juiz de For a, Juiz de For a, Brazil; 4 Graduate Program in Physical Education, Federal University of Vale do São Francisco, Petrolina, Brazil; 5 Graduate Program in Physical Education, State University of Maringá, Maringá, Brazil; 6 Graduate Program in Physical Education, State University of Londrina, Londrina, Brazil; Iwate Medical University, JAPAN

## Abstract

The objective of the current study was to compare the effects of resistance training frequency on cognitive inhibitory control in young adults with previous experience in the modality. Male participants (*N* = 36) were randomly placed into one of three experimental groups. Participants performed resistance training 1 (F1), 2 (F2), and 3 (F3) times per week for 24 weeks. The three groups performed exercises of equal intensity, volume-load, and rest duration. Cognitive inhibitory control (via Stroop test) was tested 72 h before (pre-experiment) and 72 h after (post-experiment) the resistance training program. No time vs. group interaction effects were noted for accuracy (*F*_(4, 29)_ = 3.57, *p* = 0.18) or response time (*F*_(4, 29)_ = 2.61, *p* = 0.06) on the Stroop test. These results indicate that increased resistance training frequency, when volume-load is kept constant, does not appear to potentiate cognitive inhibitory control.

## Introduction

Cognitive function corresponds to an intellectual process in which an individual becomes aware of perceiving or understanding ideas [1]. Cognitive function is composed of attention, memory, inhibitory control, and cognitive flexibility [1]. Improved cognitive function is thought to result in faster and more accurate information processing, and a delay in cerebral aging [2]. Further, research indicates that young adults with low levels of physical activity present lower cognitive function scores when compared to active individuals (i.e., those participating in sports or regular exercise) [1].

Previous studies have revealed that physical exercise might improve cognitive function [3–4]. One of the most popular types of exercise among young adults is resistance training, which can be defined as muscular contractions against external resistance, typically using machines or free weights [5]. Variables to consider when performing resistance training include intensity zone (i.e., the area being trained), duration of rest, speed of execution, duration under tension, frequency of sessions, number of repetitions, and number of sets [6]. Intensity zone, duration of rest, and speed of execution comprise the intensity component of resistance training [7], whereas frequency, number of sets, and number of repetitions are considered part of the volume component [5].

In young adults, academic and work demands may limit the frequency of physical exercise. However, frequency may be a useful variable to consider if the aim of the individual is to improve cognitive function. Specifically, resistance training frequency is defined as the number of sessions per week or the number of sessions for the same muscle group [8]. Although young adults may have only a few days for training each week, it might be worth prioritizing three sessions of resistance exercise to improve cognitive inhibitory control. This is because resistance training may produce a positive balance of protein synthesis [8] and increase brain-derived neurotrophic factor (BDNF), which is thought to be primarily responsible for exercise-related improvements in cognitive function [9].

From a practical viewpoint, changes in inhibitory control due to a manipulation of exercise frequency may ease the decision-making of exercise science professionals. Hence, the aim of the current study was to compare the effects of resistance training frequency on inhibitory control in young adults with previous experience in the modality. Our hypothesis was that increased frequency of resistance training would result in enhanced inhibitory control.

## Materials and methods

### Participants

A sample size calculation was conducted by G*Power 3.1, with a set power of 0.90, α = 0.05, and effect size of 0.35. Results indicated 30 subjects were necessary to perform the study. However, to account for possible dropouts, an additional 20% were recruited, resulting in a total of 36 participants.

Male participants (*N* = 36), aged 18–30 years, were selected in a non-probabilistic way. Participants had performed resistance training for at least five consecutive years, did not present any history of injury, and had not used any kind of ergogenic for strength, muscular volume, or cognitive performance in the last year. Researchers asked participants to maintain their daily routines (e.g., eating habits), and to only engage in our exercise program.

After receiving information about the procedures, all participants provided written informed consent. The study protocol was approved by the Ethics Committee of the Universidade Federal de Pernambuco (CAAE—47571415.9.0000.5208) according to the standards set by the Declaration of Helsinki.

### Experimental design

The current study was a randomized, controlled experimental investigation, with a 24-week duration, and carried out in male young adults with previous experience in resistance training.

**Fig 1:**
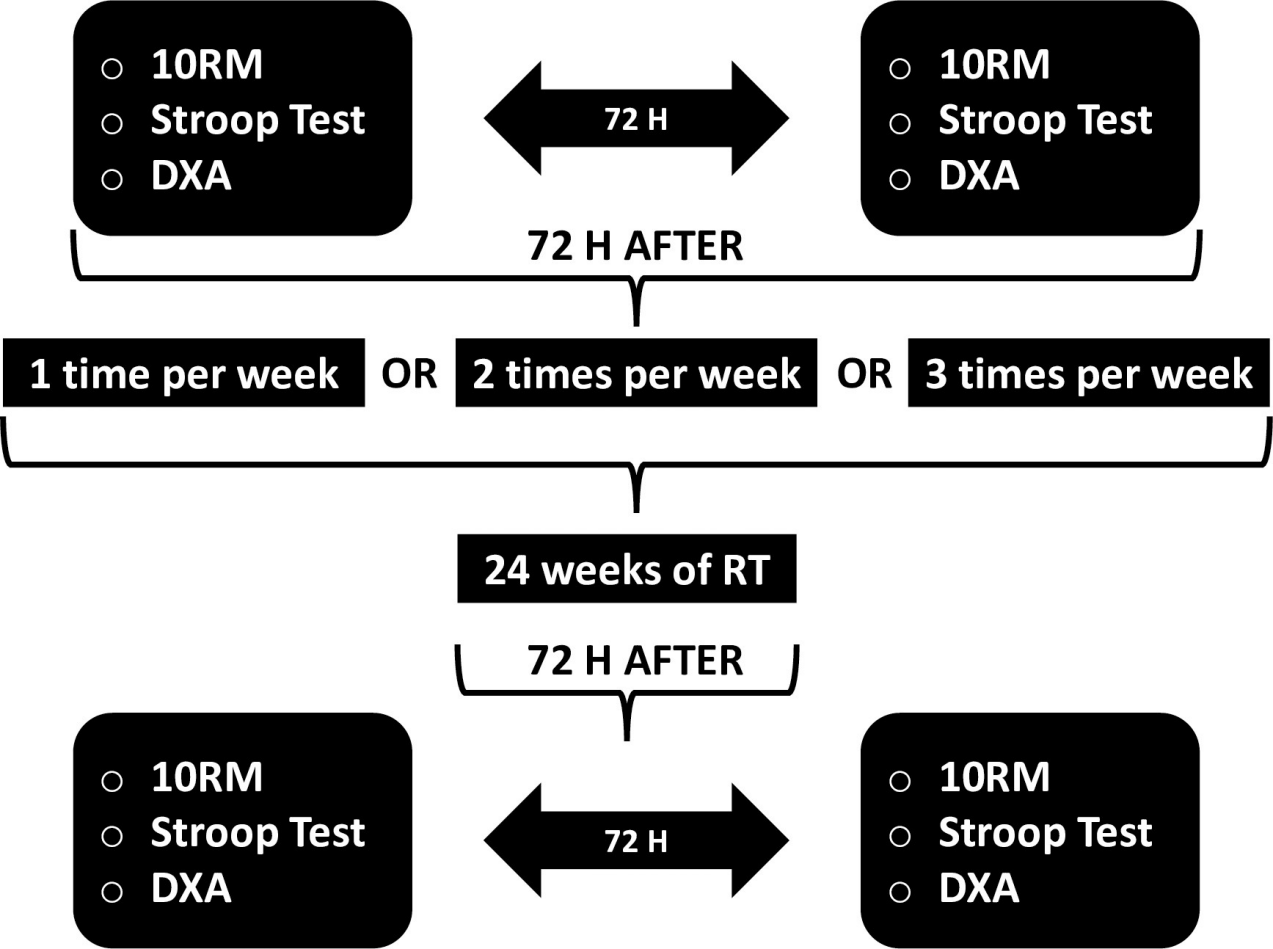
Experimental design of investigation. *Note*. RM = repetition maximum; DXA = dual-energy x-ray absorptiometry; RT = resistance training.

Participants underwent the 24-week investigation (Fig 1). Order of dependent measures were counterbalanced [inhibitory control performance and 10 repetition maximum test (10 RM)] across three experimental groups: one (F1), two (F2), and three (F3) resistance training sessions per week. The groups performed the same exercises, with equalized intensity (load zone), load volume [repetitions x load (kg)], and rest duration (Table 1).

**Table 1:** Resistance training program.

Weeks	1 session per week	2 sessions per week	3 sessions per week
1 to 24	6 sets 10 RM; 180 s between sets and exercise	3 sets 10 RM; 180 s between sets and exercise	2 sets 10 RM; 180 s between sets and exercise

*Note*. RM = repetition maximum.

In addition, 10 RM was conducted to calculate the intensity zone that was adjusted weekly (increased/decreased 2–5% for upper and 5–10% for lower limbs), according Schoenfeld et al. [5]. If the participant performed more than 10 repetitions in at least two consecutive experimental sessions, the load (kg) was increased in the following session, whereas if the participant was unable to perform ten repetitions, the load (kg) was decreased in the subsequent session.

The Stroop Test assessed inhibitory control 72 h before (pre-experiment) and 72 h after (post-experiment) the resistance training program. Additionally, participants refrained from any physical exercise for 48 h prior to the inhibitory control assessment.

### Resistance training program

The resistance training program utilized in the current study was based on previous guidelines for healthy young adults [6] and was composed by six exercises (bench press, leg press 45^o^, seated row, squat, shoulder press, and leg curl) performed one (Monday), two (Monday and Wednesday), or three times per week (Monday, Wednesday, and Friday) during 24 consecutive weeks. The sessions were conducted at the same time of the day (4 p.m.) to avoid any circadian rhythm influence. In addition, two experienced researchers supervised all sessions.

Table 1 shows the training program performed by each experimental group (1 vs. 2 vs. 3 sessions per week). Participants warmed-up (1 x 15–20 repetitions with 50% of predicted 10 RM and 1 x 10–15 repetitions with 70% of predicted 10 RM; 3 min interval between sets) for the first upper and lower limb exercises (bench press and leg press 45^o^) [7].

### Variable measurements

#### Cognitive function

We adopted the Stroop Test [10] to assess inhibitory control 72 h before and after testing period. Additionally, we adopted the score means as baseline, following Bruce et al. [11] recommendations. Intraclass correlation coefficients and standard error of the measurement between the two baseline inhibitory control assessments were 0.94 and 3.2% for accuracy; 0.97 and 0.09 ms for response time, respectively. The Stroop Test was conducted on a high-definition laptop (MacBook Pro, A1502 model, EUA; 1800 x 1260 pixels). In the test, participants answered the physical color of color words. The physical colors of the words may be different from the color denoted by the actual word (e.g. the word “green” might be yellow in color, or the word “red” might be blue in color). A total of 62 words, with a 200 ms interval between the response and a new stimulus, were presented. Moreover, stimulus did not fade from the screen until a response was given. Stimuli varied between congruent (word and color have the same meaning), incongruent (word and color have different meaning), and control (colored rectangle with one of the colors of the test (i.e., red, green, blue, or black). The participants pressed the D (red), F (green), J (blue), and K (black) keys to respond. When the answer was correct, the stimulus faded and a new stimulus was provided. In case of incorrect answers, an “X” showed up on the screen and a new stimulus appeared subsequently. At the end of the test, scores for accuracy, mean response time, and errors were collected. All participants had total access to their test results. Evaluator was trained to administer the test and was blind to group for all assessments. All participants were familiar with the Stroop Test prior to data collection, as one week prior to the first baseline Stroop test, participants performed three sets (congruent, incongruent, and control) with 10 words each and 200 ms interval between the response and a new stimulus.

#### 10 RM

Intensity zone for 10 RM was determined following the 10 RM test. The exercises performed were bench press, leg press 45^o^, seated row, squat, shoulder press, and leg curl.

Participants performed the 10 RM test in two distinct sections, with intervals of 72 h. For each exercise, two attempts were performed with intervals of 10 minutes between sets and exercises. Intraclass correlation coefficients and standard error of the measurement between familiarization and 10 RM were 0.97 and 3.2 kg for bench press, 0.98 and 4.6 kg for leg press 45°, 0.98 and 3.1 kg for seated row, 0.99 and 2.2 kg for squat, 0.97 and 1.6 kg for shoulder press, and 0.99 and 2.5 kg for leg curl, respectively.

A warm-up (2 x 15–20 reps with 50% of predicted 1 RM, with 120 second intervals between sets) was carried out for each exercise before the 10 RM. Verbal encouragement was given throughout the 10 RM.

#### Body composition

Total body mass (kg–portable scale PL 200, Filizola S.A., Sao Paulo, Brazil, accuracy of 0.1 kg) and height (professional stadiometer Sanny, Sao Paulo, Brazil, accuracy of 0.1 cm) were measured. Corporal density was measured using the technique of body scanning by Dual X-ray Absorptiometry (DXA; Hologic, Waltham, MA). Participants were recommended to not performing any physical activity for at least 48 h and ad libitum hydration the day before the test. Participants remained in the supine position, with the arms next to their body, and hands in neutral position. Feet and knees were 10 cm away and tied with a Velcro band to avoid any movement that might interfere with image visualization during the procedure. The analyzed variables were: free fat mass, fat mass, and total body mass. DXA calibration followed the manufacturer recommendations. Additionally, measurements were performed by an experienced evaluator who was blind to experimental condition.

### Data analysis

Shapiro-Wilk and Levene tests evaluated normality and homoscedasticity of the data, respectively. A one-way analysis of variance (ANOVA) was used to determine differences (inhibitory control, 10 RM, total body mass, non-fat mass, and fat mass) between groups at baseline. A two-way ANOVA was used to determine the interaction between time (pre vs post) and group (F1 vs F2 vs F3) for inhibitory control, 10 RM, non-fat mass, and fat mass. In addition, when necessary, a Bonferroni post hoc test was used to determine statistical differences. Moreover, effect size was used to analyze differences from a practical point of view. Thereby, according to Rhea [12], the following criteria was adopted: *h*^*2*^ < 0.35 = trivial, 0.35 ≤ *h*^*2*^ < 0.8 = small effect size, 0.8 ≤ *h*^*2*^ < 1.5 = moderate effect size and, *h*^*2*^ ≥ 1.5 = large effect size. Data were analyzed via SPSS 21.0, and *p* < .05 was considered statistically significant.

## Results

**Fig 2:**
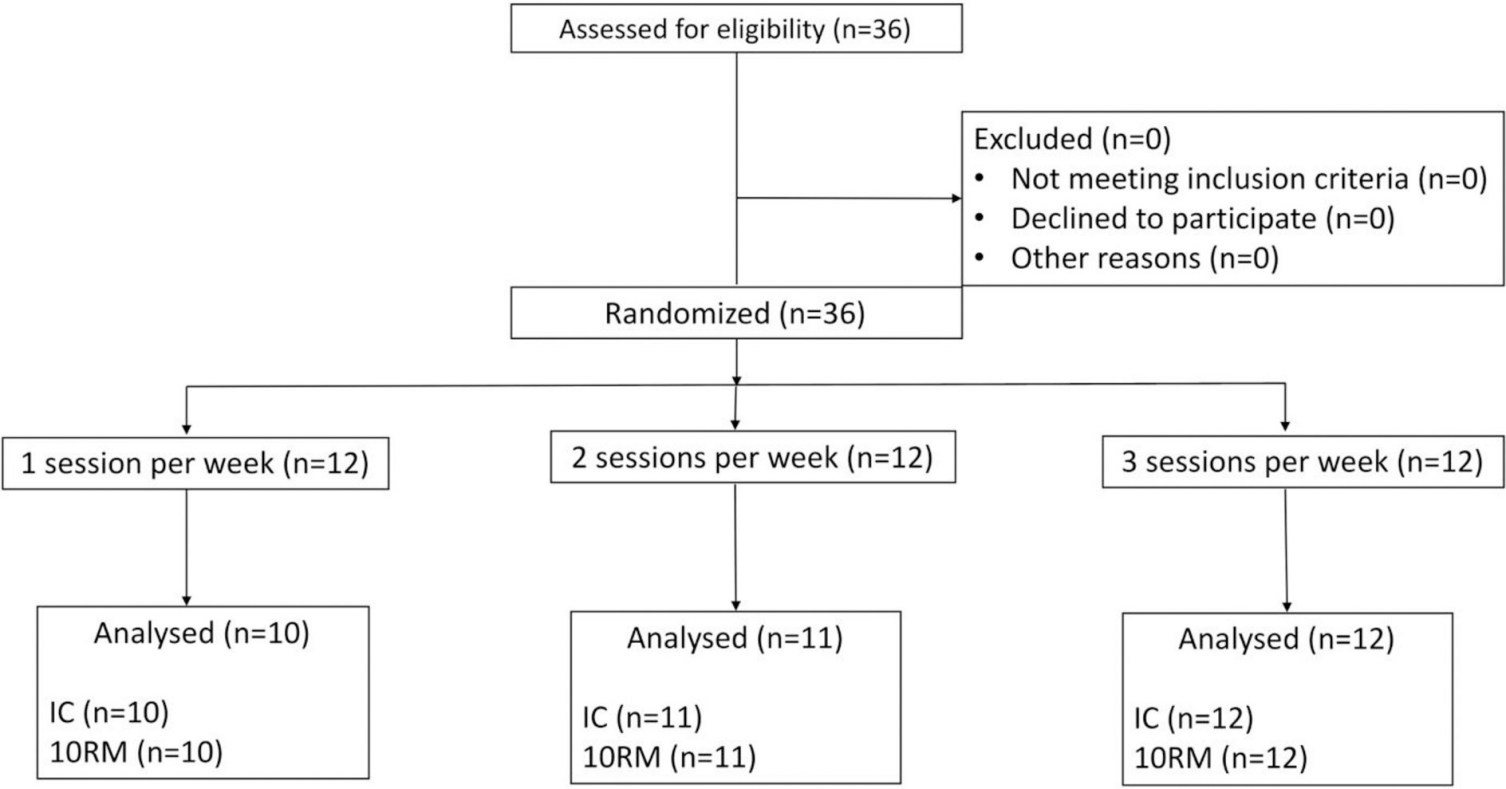
Flowchart of the analyzed participants. *Note*. IC = Inhibitory control; RM = repetition maximum.

A total of 33 of the original 36 participants finished the entire 24-week study (Fig 2). Age, inhibitory control, 10 RM, non-fat mass, fat mass, and total body mass baseline values are shown in Table 2.

**Table 2:** Descriptive values (mean and standard deviation) of the variables at baseline.

Variables	Baseline	
	Mean	SD
Age (years)	22.1	3.8
Stroop Test Accuracy (%)	87.4	10.2
Stroop Test Latency (ms)	661.6	149.8
10 RM bench press (kg)	62.7	11.4
10 RM leg press 45^o^ (kg)	165.8	24.7
10 RM seated row (kg)	54.6	9.2
10 RM squat (kg)	114.0	18.3
10 RM shoulder press (kg)	11.3	4.2
10 RM leg curl (kg)	57.1	10.5
Non-fat mass (kg)	67.9	9.3
Fat mass (kg)	13.4	7.9
Total body mass (kg)	81.2	10.8

*Note*. RM = repetition maximum; SD = standard deviation.

### Baseline

All groups (F1, F2, and F3) were similar at baseline for accuracy [*F*_(3, 33)_ = 3.02, *p* = 0.23], response time [*F*_(3, 33)_ = 2.14, *p* = 0.29], 10 RM in bench press [*F*_(3, 33)_ = 1.67, *p* = 0.37], leg press 45^o^ [*F*_(3, 33)_ = 2.65, *p* = 0.17], seated row [*F*_(3, 33)_ = 2.40, *p* = 0.19], squat [*F*_(3, 33)_ = 3.59, *p* = 0.13], shoulder press [*F*_(3, 33)_ = 3.57, *p* = 0.18], leg curl [*F*_(3, 33)_ = 1.46, *p* = 0.36], non-fat mass [*F*_(3, 33)_ = 3.11, *p* = 0.41], fat mass [*F*_(3, 33)_ = 2.69, *p* = 0.42], and total body mass [*F*_(3, 33)_ = 1.98, *p* = 0.54].

### Volume-load

The mean volume-load during the training period were similar among the testing groups [F1 = 590.402 ± 87.231 kg; F2 = 537.745 ± 74.769 kg; F3 = 558.281 ± 96.114 kg; *F*_(3, 33)_ = 1.98, *p* = 0.46].

### Inhibitory control

**Fig 3:**
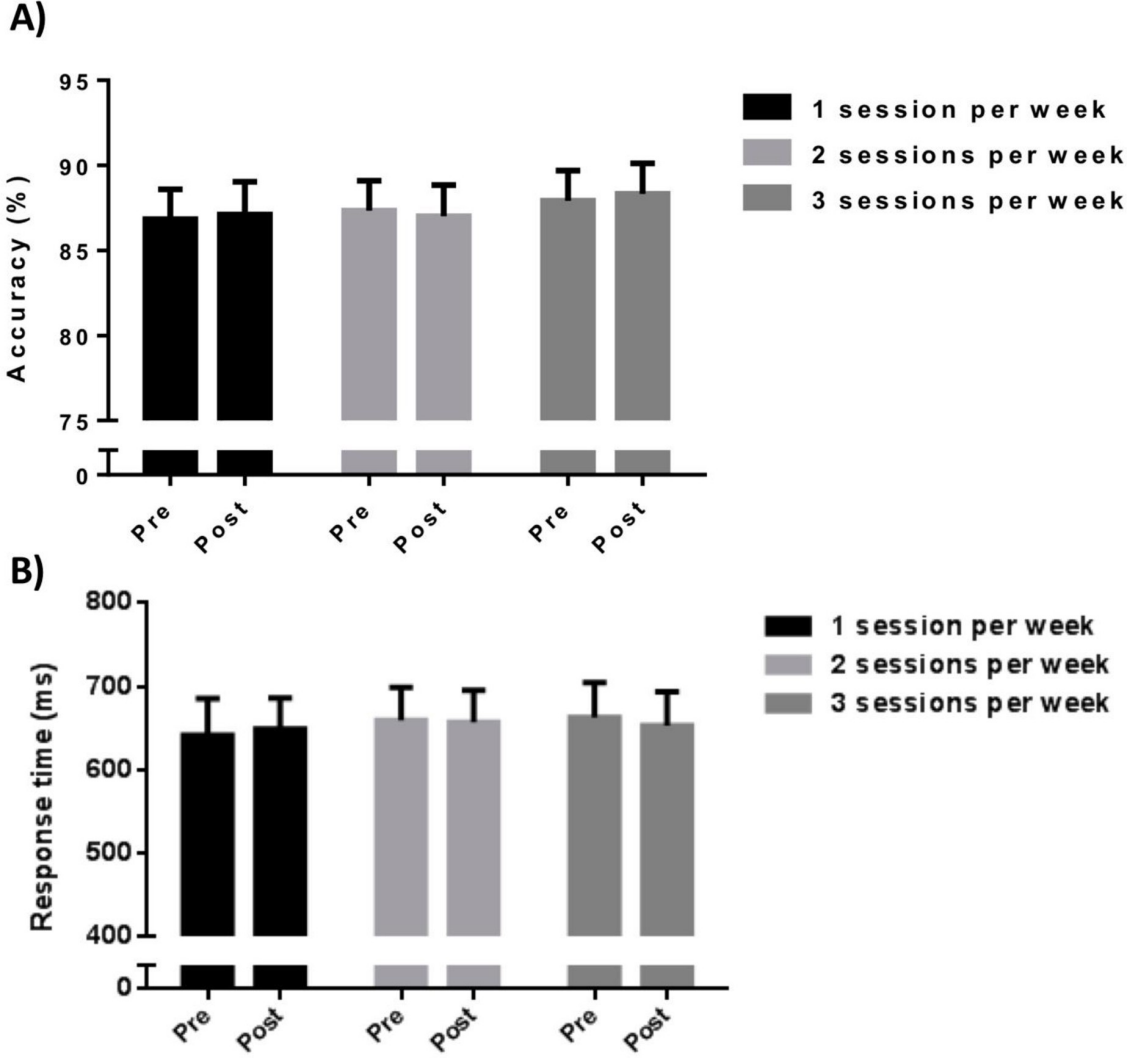
(A and B). Inhibitory control findings according to group (F1 vs. F2 vs. F3).

For accuracy, there was no a significant main effect for time [*F*_(2, 31)_ = 1.69, *p* = 0.32] or group [*F*_(3, 30)_ = 2.14, *p* = 0.27]. Additionally, no interaction was observed (time vs. group) for accuracy [*F*_(4, 29)_ = 3.57, *p* = 0.18; Fig 3A, Table 3]. Similarly, For response time, there was no a significant main effect for time [*F*_(2, 31)_ = 2.68, *p* = 0.22] or group [*F*_(3, 30)_ = 2.05, *p* = 0.36]. Furthermore, no interaction was observed (time vs. group) for response time [*F*_(4, 29)_ = 2.61, *p* = 0.06; Fig 3B, Table 3].

**Table 3:** Mean and standard deviation of cognitive function (accuracy and response time) according to group (F1 vs. F2 vs. F3) and time (pre vs. post).

Variables	F1 (*n* = 10)	F2 (*n* = 11)	F3 (*n* = 12)	Effects	*F*	*p*
Accuracy (%)						
Pre	86.3 ± 9.6	87.8 ± 10.6	88.1 ± 10.2			
Post	87.1 ± 11.4	87.2 ± 11.9	88.5 ± 12.5	Interaction		
Δ%	1.3 ± 0.4	0.9 ± 0.6	1.4 ± 0.8	T x G	3.57	0.18
ES	0.1	0.1	0.2			
Response time (ms)						
Pre	673.5 ± 148.9	664.9 ± 117.6	652.4 ± 123.0			
Post	648.6 ± 118.5	648.3 ± 118.6	647.5 ± 117.7	Interaction		
Δ%	0.8 ± 0.5	1.0 ± 0.4	1.2 ± 0.7	T x G	2.61	0.06
ES	0.1	0.1	0.1			

*Note*. F1 = 1 session per week group; F2 = 2 sessions per week groups; F3 = 3 sessions per week groups; T x G = time vs. group; Δ% = percentage change; ES = effect size.

### 10RM

There was a significant main effect of time (i.e., pre vs. post) for bench press [F1 = *F*_(2, 10)_ = 54.70, *p* = 0.01, ES = 0.5; F2 = *F*_(2, 10)_ = 43.82, *p* = 0.02, ES = 0.4; F3 = *F*_(2, 10)_ = 60.21, *p* = 0.01, ES = 0.5], leg press 45^o^ [F1 = *F*_(2, 10)_ = 65.33, *p* = 0.01, ES = 0.6; F2 = *F*_(2, 10)_ = 57.42, *p* = 0.01, ES = 0.5; F3 = *F*_(2, 10)_ = 50.18, *p* = 0.01, ES = 0.5], seated row [F1 = *F*_(2, 10)_ = 49.94, *p* = 0.02, ES = 0.3; F2 = *F*_(2, 10)_ = 56.31, *p* = 0.01, ES = 0.4; F3 = *F*_(2, 10)_ = 53.90, *p* = 0.01, ES = 0.4], squat [F1 = *F*_(2, 10)_ = 68.03, *p* = 0.01, ES = 0.7; F2 = *F*_(2, 10)_ = 71.45, *p* = 0.01, ES = 0.6; F3 = *F*_(2, 10)_ = 53.28, *p* = 0.03, ES = 0.4], shoulder press [F1 = *F*_(2, 10)_ = 36.3, *p* = 0.03, ES = 0.3; F2 = *F*_(2, 10)_ = 44.09, *p* = 0.02, ES = 0.3; F3 = *F*_(2, 10)_ = 41.44, *p* = 0.01, ES = 0.4] and leg curl [F1 = *F*_(2, 10)_ = 85.1, *p* = 0.01, ES = 0.7; F2 = *F*_(2, 10)_ = 73.46, *p* = 0.01, ES = 0.6; F3 = *F*_(2, 10)_ = 89.42, *p* = 0.01, ES = 0.7]. However, there was no significant main effect of group for bench press [*F*_(3, 30)_ = 1.67, *p* = 0.40], leg press 45^o^ [*F*_(3, 30)_ = 2.17, *p* = 0.27], seated row [*F*_(3, 30)_ = 1.33, *p* = 0.56], squat [*F*_(3, 30)_ = 1.19, *p* = 0.45], shoulder press [*F*_(3, 30)_ = 1.71, *p* = 0.34], or leg curl [*F*_(3, 30)_ = 2.07, *p* = 0.25]. The findings did not indicate and interaction (time vs. group) for bench press [*F*_(4, 29)_ = 2.56, *p* = 0.17], leg press 45^o^ [*F*_(4, 29)_ = 1.58, *p* = 0.35], seated row [*F*_(4, 29)_ = 2.01, *p* = 0.28], squat [*F*_(4, 29)_ = 1.16, *p* = 0.42], shoulder press [*F*_(4, 29)_ = 2.42, *p* = 0.32], or leg curl [*F*_(4, 29)_ = 3.79, *p* = 0.14; Table 4].

**Table 4:** Ress1ults of 10 RM (main effects and ES).

Exercise	F1 (*n* = 10)	F2 (*n* = 11)	F3 (*n* = 12)	Effects	F	*p*
Bench Press						
Pre	60.8 ± 9.6	63.5 ± 12.0	61.3 ± 10.3	Time	46.61	0.001
Post	64.0 ± 11.8	66.2 ± 10.3	65.9 ± 12.5	Group	1.67	0.40
Δ%	5.1 ± 2.3	4.2 ± 1.9	4.8 ± 2.7	T x G	2.56	0.17
ES	0.5	0.4	0.5			
Leg Press 45^o^						
Pre	161.8 ± 23.5	167.2 ± 27.8	168.9 ± 25.6	Time	54.70	0.001
Post	173.6 ± 27.9	176.7 ± 24.1	175.4 ± 30.3	Group	2.17	0.27
Δ%	7.3 ± 3.0	6.1 ± 2.8	5.6 ± 2.5	T x G	1.58	0.35
ES	0.6	0.5	0.5			
Seated row						
Pre	55.9 ± 8.6	52.0 ± 9.9	51.7 ± 9.3	Time	50.87	0.001
Post	57.4 ± 9.2	55.7 ± 10.3	54.0 ± 8.5	Group	1.33	0.56
Δ%	3.0 ± 2.4	4.8 ± 2.6	4.2 ± 1.9	T x G	2.01	0.28
ES	0.3	0.4	0.4			
Squat						
Pre	112.3 ± 20.1	108.6 ± 16.4	116.7 ± 17.8	Time	64.99	0.001
Post	120.6 ± 23.7	114.9 ± 19.4	121.1 ± 17.5	Group	1.19	0.45
Δ%	8.3 ± 3.7	6.5 ± 3.0	5.5 ± 2.8	T x G	1.16	0.42
ES	0.7	0.6	0.4			
Shoulder Press						
Pre	11.0 ± 3.4	10.3 ± 4.5	11.9 ± 3.9	Time	42.84	0.01
Post	11.5 ± 2.7	10.7 ± 4.2	12.4 ± 3.1	Group	1.71	0.34
Δ%	3.7 ± 1.8	3.2 ± 1.5	4.0 ± 2.1	T x G	2.42	0.32
ES	0.3	0.3	0.4			
Leg Curl						
Pre	59.4 ± 9.2	60.2 ± 11.6	56.7 ± 8.7	Time	78.6	0.001
Post	65.3 ± 10.5	64.7 ± 11.4	62.8 ± 9.0	Group	2.07	0.25
Δ%	9.3 ± 4.5	8.0 ± 3.9	10.2 ± 4.8	T x G	3.79	0.14
ES	0.7	0.6	0.7			

*Note*. F1 = 1 session per week group; F2 = 2 sessions per week groups; F3 = 3 sessions per week groups; T x G = time vs. group; Δ% = percentage change; ES = effect size.

### Body composition

Calculation of body composition revealed no significant main effect of time for non-fat mass [*F*_(2, 31)_ = 2.36, *p* = 0.24], fat mass [*F*_(2, 31)_ = 1.99, *p* = 0.40], or total body mass [*F*_(2, 31)_ = 1.13, *p* = 0.55]. Additionally, there was no a significant main effect of group for non-fat mass [*F*_(3, 30)_ = 1.67, *p* = 0.40], fat mass [*F*_(3, 30)_ = 1.24, *p* = 0.30], or total body mass [*F*_(3, 30)_ = 1.23, p = 0.51]. Finally, results indicated no interaction (time vs. group) for non-fat mass [*F*_(4, 29)_ = 2.34, *p* = .020], fat mass [*F*_(4, 29)_ = 2.13, *p* = 0.23], or total body mass [*F*_(4, 29)_ = 3.19, *p* = 0.33].

## Discussion

The aim of the current study was to determine the effect of resistance training frequency on cognitive inhibitory control in young adults with previous experience in the modality. Results indicated no differences among the three testing groups. In other words, if variables related to volume are equalized, inhibitory control response remains the same, regardless of frequency.

Our findings indicate inhibitory control performance (i.e., accuracy and response time) remained the same in the three groups. However, other studies have shown improvement in cognitive function following resistance training [13–14]. These disparate results may be due to previous investigations recruiting physically inactive or moderately active participants. Although resistance training might improve cognitive function in individuals who do not typically perform this type of exercise, experienced participants may not receive the same increase in function. In other words, the more the individuals performs resistance training, the smaller the increase in cognitive function, representing a possible ceiling effect. Additionally, previous research has shown that resistance training volume can positively influence inhibitory control [15]. However, in the present study, volume was equalized among the groups. This may explain the performance similarity on the Stroop Test (i.e., accuracy and response time). Thus, the number of contractions performed during an exercise program may be associated with cognitive function through the mechanism of augmented blood circulation, which improves BDNF circulation [1]. If this is the case, an increase in frequency of resistance training, but maintaining the same weekly volume may be a poor strategy to improve inhibitory control in young adults.

According to muscular strength, the groups improved performance in every exercise, although, between groups they were similar. Despite the systematic review by Schoenfeld et al., [8] which indicated dose-dependent response of training frequency on muscular hypertrophy, it seems the same phenomenon is not observable for muscular strength. Nevertheless, the same systematic review included several studies that did not equalize training volume, even though the purpose of the study was to analyze the effect of training frequency of resistance training on muscular strength. Thus, considering the improvement on muscular strength in the three experimental groups, our study corroborates studies conducted by Fonseca et al. [7] and Schoenfeld et al. [5] that demonstrated resistance training program with a load zone of 10 RM improves muscular strength. In short, considering the findings of the present study for the young men trained, if the aim is to improve 10 RM (e.g., bench press, leg press 45^o^, seated row, squat, shoulder press, and/or leg curl), the frequency of resistance training (i.e., 1, 2, or 3 times per week) does not appear to matter, if the weekly volume-load is the same.

Concerning body composition, our results indicate no dose-dependent effect of resistance training frequency on non-fat mass, fat mass, or total body mass. These findings diverge from other studies [5, 16–17]. As such, it is reasonable to point out the main differences among these investigations. First, the duration of previous studies was shorter when compared to the present study (i.e., 24 weeks). Second, participants in previous research were moderately active, whereas, in the current study, they were highly trained. The more trained the participant, the less increase can be expected in the components of body composition (e.g., non-fat mass, fat mass, and total body mass) [12].

Although the present study revealed interesting findings that adds crucial information to the literature, it presents some limitations. We did not utilize magnetic resonance; therefore, we lack images that could indicate any physiological changes in inhibitory control. Moreover, the lack of analysis of brain electrical activity (brain waves) may also indicate another limitation. Further BDNF levels and inflammatory biomarkers were also not analyzed. Thus, the current findings should be considered with caution. Nonetheless, strengths of our research include the length of 24-week experiment, and the two Stroop Test baseline measurements, reducing random error of the neurocognitive measures found in Bruce et al. [11].

## Conclusion

It seems that frequency of resistance training may not cause brain neurogenesis [18], defined as the production of new neurons [19]. Thus, augmented frequency, when the volume is equalized, should not be considered by training center professionals if the aim is to improve cognitive inhibitory control, strength, or body composition in trained young adults. From a practical viewpoint, independent of the number of sessions per week, weekly volume should be more heavily considered. Hence, the findings of the study revealed crucial parameters in exercise prescription for exercise science professionals, especially athletes.

## Supporting information

S1 File Supporting information. Figure A. Baseline. Figure B. Post-intervention.
(DOCX)
