# Supplementary material for: Frequency of resistance training does not affect inhibitory control or improve strength in well-trained young adults
Source: PLoS One. 2018 Nov 2;13(11):e0206784. doi: 10.1371/journal.pone.0206784 (PMC6214533; doi:10.1371/journal.pone.0206784)
Supplement: S1 File — Figure A. Baseline. Figure B. Post-intervention. (DOCX) [file pone.0206784.s001.docx]

**Supporting Information**

Figure A. Baseline

| 1 vez por semana | |  |  |  | 2 vezes por semana | |  |  |  | 3 vezes por semana | |  |
| --- | --- | --- | --- | --- | --- | --- | --- | --- | --- | --- | --- | --- |
| Idade | Accuracy (%) | Response time (ms) |  |  | Idade | Accuracy (%) | Response time (ms) |  |  | Idade | Accuracy (%) | Response time (ms) |
|  | 81 | 823 |  |  |  | 83 | 786 |  |  |  | 78 | 823 |
|  | 100 | 451 |  |  |  | 97 | 504 |  |  |  | 95 | 667 |
|  | 93 | 562 |  |  |  | 96 | 542 |  |  |  | 93 | 763 |
|  | 84 | 799 |  |  |  | 84 | 784 |  |  |  | 100 | 678 |
|  | 87 | 706 |  |  |  | 85 | 742 |  |  |  | 91 | 605 |
|  | 91 | 502 |  |  |  | 90 | 606 |  |  |  | 85 | 707 |
|  | 80 | 887 |  |  |  | 82 | 761 |  |  |  | 84 | 634 |
|  | 88 | 833 |  |  |  | 88 | 450 |  |  |  | 74 | 653 |
|  | 78 | 624 |  |  |  | 81 | 731 |  |  |  | 86 | 745 |
|  | 92 | 571 |  |  |  | 92 | 680 |  |  |  | 91 | 654 |
|  | 89 | 783 |  |  |  | 89 | 624 |  |  |  | 93 | 569 |
|  | 79 | 542 |  |  |  | 81 | 769 |  |  |  | 85 | 331 |
|  | 93 | 562 |  |  |  | 96 | 542 |  |  |  | 93 | 763 |
|  | 91 | 502 |  |  |  | 90 | 606 |  |  |  | 85 | 707 |
|  | 88 | 833 |  |  |  | 88 | 450 |  |  |  | 74 | 653 |
|  | 92 | 571 |  |  |  | 92 | 680 |  |  |  | 91 | 654 |
|  | 81 | 823 |  |  |  | 83 | 786 |  |  |  | 78 | 823 |
|  | 100 | 451 |  |  |  | 97 | 504 |  |  |  | 95 | 667 |
|  | 78 | 624 |  |  |  | 81 | 731 |  |  |  | 86 | 745 |
|  | 89 | 783 |  |  |  | 89 | 624 |  |  |  | 93 | 569 |
|  | 84 | 799 |  |  |  | 84 | 784 |  |  |  | 100 | 678 |
|  | 80 | 887 |  |  |  | 82 | 761 |  |  |  | 84 | 634 |
|  | 79 | 542 |  |  |  | 81 | 769 |  |  |  | 85 | 331 |
|  | 100 | 403 |  |  |  | 97 | 504 |  |  |  | 95 | 667 |
|  | 91 | 502 |  |  |  | 90 | 606 |  |  |  | 85 | 707 |
|  | 92 | 571 |  |  |  | 92 | 680 |  |  |  | 91 | 654 |
|  | 93 | 562 |  |  |  | 96 | 542 |  |  |  | 93 | 763 |
|  | 84 | 799 |  |  |  | 84 | 784 |  |  |  | 100 | 678 |
|  | 87 | 706 |  |  |  | 85 | 742 |  |  |  | 91 | 605 |
|  | 91 | 502 |  |  |  | 90 | 606 |  |  |  | 85 | 707 |
|  | 88 | 833 |  |  |  | 88 | 450 |  |  |  | 74 | 653 |
|  | 92 | 571 |  |  |  | 92 | 680 |  |  |  | 91 | 654 |
|  | 100 | 447 |  |  |  | 97 | 504 |  |  |  | 95 | 667 |
|  | 88 | 833 |  |  |  | 88 | 450 |  |  |  | 74 | 653 |
|  | 94 | 546 |  |  |  | 95 | 467 |  |  |  | 93 | 501 |
|  | 89 | 420 |  |  |  | 83 | 397 |  |  |  | 90 | 433 |

Figure B. Post-intervention

| 1 vez por semana | |  |  | 2 vezes por semana | |  |  | 3 vezes por semana | |  |
| --- | --- | --- | --- | --- | --- | --- | --- | --- | --- | --- |
| Idade | Accuracy (%) | Response time (ms) |  | Idade | Accuracy (%) | Response time (ms) |  | Idade | Accuracy (%) | Response time (ms) |
|  | 84 | 785 |  |  | 79 | 723 |  |  | 82 | 854 |
|  | 98 | 476 |  |  | 97 | 487 |  |  | 98 | 601 |
|  | 93 | 582 |  |  | 94 | 591 |  |  | 95 | 640 |
|  | 84 | 761 |  |  | 88 | 783 |  |  | 100 | 699 |
|  | 89 | 696 |  |  | 87 | 740 |  |  | 87 | 542 |
|  | 86 | 546 |  |  | 93 | 576 |  |  | 89 | 762 |
|  |  |  |  |  |  |  |  |  | 81 | 653 |
|  | 88 | 844 |  |  | 88 | 485 |  |  | 79 | 702 |
|  | 81 | 638 |  |  | 81 | 768 |  |  | 80 | 690 |
|  | 89 | 570 |  |  | 86 | 613 |  |  | 94 | 661 |
|  |  |  |  |  | 84 | 562 |  |  | 90 | 587 |
|  | 79 | 588 |  |  | 80 | 804 |  |  | 85 | 379 |
|  | 98 | 476 |  |  | 97 | 487 |  |  | 98 | 601 |
|  | 86 | 546 |  |  | 93 | 576 |  |  | 89 | 762 |
|  | 81 | 638 |  |  | 81 | 768 |  |  | 80 | 690 |
|  | 89 | 570 |  |  | 86 | 613 |  |  | 94 | 661 |
|  | 84 | 785 |  |  | 79 | 723 |  |  | 82 | 854 |
|  | 93 | 582 |  |  | 94 | 591 |  |  | 95 | 640 |
|  | 89 | 696 |  |  | 87 | 740 |  |  | 87 | 542 |
|  | 88 | 844 |  |  | 88 | 485 |  |  | 79 | 702 |
|  | 79 | 588 |  |  | 80 | 804 |  |  | 85 | 379 |
|  | 86 | 546 |  |  | 93 | 576 |  |  | 89 | 762 |
|  | 89 | 570 |  |  | 86 | 613 |  |  | 94 | 661 |
|  | 93 | 582 |  |  | 94 | 591 |  |  | 95 | 640 |
|  | 93 | 582 |  |  | 94 | 591 |  |  | 95 | 640 |
|  | 89 | 696 |  |  | 87 | 740 |  |  | 87 | 542 |
|  | 88 | 844 |  |  | 88 | 485 |  |  | 79 | 702 |
|  | 89 | 570 |  |  | 86 | 613 |  |  | 94 | 661 |
|  | 84 | 785 |  |  | 79 | 723 |  |  | 82 | 854 |
|  | 84 | 761 |  |  | 88 | 783 |  |  | 100 | 699 |
|  |  |  |  |  |  |  |  |  | 81 | 653 |
|  | 89 | 570 |  |  | 86 | 613 |  |  | 94 | 661 |
|  | 98 | 476 |  |  | 97 | 487 |  |  | 98 | 601 |
|  | 84 | 761 |  |  | 88 | 783 |  |  | 100 | 699 |
|  | 86 | 546 |  |  | 93 | 576 |  |  | 89 | 762 |
|  | 88 | 844 |  |  | 88 | 485 |  |  | 79 | 702 |
